# Potential mitigation of environmental impacts of intensive plum production in southeast China with maintenance of high yields: Evaluation using life cycle assessment

**DOI:** 10.3389/fpls.2023.1158591

**Published:** 2023-03-22

**Authors:** Xiaojun Yan, Delian Ye, Yafu Tang, Muhammad Atif Muneer, Peter Christie, Congyue Tou, Weidong Xu, Bingrong Shen, Jinxian Xu, Jiangzhou Zhang

**Affiliations:** ^1^ International Magnesium Institute, College of Resources and Environment, Fujian Agriculture and Forestry University, Fuzhou, China; ^2^ College of Resources and Environmental Sciences, China Agricultural University, Beijing, China; ^3^ Soil and Fertilizer Station of Zhaoan County, Zhangzhou, China

**Keywords:** *Prunus salicina* Lindl., environmental impacts, nutrient efficiency, life cycle assessment (LCA), nitrogen management

## Abstract

**Introduction:**

Intensive plum production usually involves high yields but also high environmental costs due to excessive fertilizer inputs. Quantitative analysis of the environmental effects of plum production is thereby required in the development of optimum strategies to promote sustainable fruit production.

**Methods:**

We collected survey questionnaires from 254 plum production farms in Zhao’an county, Fujian province, southeast China to assess the environmental impacts by life cycle assessment (LCA) methodology. The farms were categorized into four groups based on yield and environmental impacts, i.e., LL (low yield and low environmental impact), LH (low yield but high environmental impact), HL (high yield but low environmental impact), and HH (high yield and high environmental impact).

**Results:**

The environmental impacts, i.e., average energy depletion, global warming, acidification, and eutrophication potential in plum production were 18.17 GJ ha^-1^, 3.63 t CO_2_ eq ha^-1^, 42.18 kg SO_2_ eq ha^-1^, and 25.06 kg PO_4_ eq ha^-1^, respectively. Only 19.7% of farmers were in the HL group, with 13.3% in the HH group, 39.0% in LL, and 28.0% LH. Plum yields of the HL group were 109-114% higher than the mean value of all 254 farms. Additionally, the HL group had a lower environmental impact per unit area compared to the overall mean value, with a reduction ranging from 31.9% to 36.7%. Furthermore, on a per tonne of plum production basis, the energy depletion, global warming potential, acidification potential, and eutrophication potential of HL farms were lower by 75.4%, 75.0%, 75.6%, and 75.8%, respectively. Overall, the total environmental impact index of LL, LH, HL, and HH groups were 0.26, 0.42, 0.06, and 0.21, respectively.

**Discussion:**

Excessive fertilizer N application was the main source of the environmental impacts, the potential to reduce fertilizer N rate can be achieved without compromising plum yield by studying the HH group. The results provide an important foundation for enhancing the management of plum production, in order to promote ‘green’ agricultural development by reducing environmental impacts.

## 1 Introduction

Plum (*Prunus salicina* Lindl.), commonly known as Japanese or Chinese plum, is an economically important fruit crop in China with a cultivated area of 211×10^4^ ha and an annual production of 700 × 10^4^ tonnes, accounting for 55.6% of world plum production in 2019 (FAO, 2021). During the past thirty years the yield of plum per unit area has increased by 102.8% in China, mainly through the application of synthetic fertilizers (Carranca et al., 2018). Orchard fruits provide high economic returns and there is no guidance to farmers on appropriate fertilizer application rates. The resulting overuse of synthetic fertilizers is of great concern because of the implications for agricultural sustainability and the health of the environment (Li et al., 2019; Chen et al., 2020). Research by Zhang et al. (2020) found that 97% of orchards exhibit nitrogen surplus, which underscores the importance of proper fertilizer management to achieve sustainable orchard production with minimal environmental damage (Shah and Wu, 2019). Hence, comprehending and mitigating the probable environmental impacts of intensive plum production is crucial.

Life cycle assessment (LCA) is a commonly used tool for assessment of the potential environmental impacts of products, processes, or activities (Loiseau et al., 2018). Energy depletion and global warming potential are considered key factors related to environmental impacts on agricultural production systems (Chen et al., 2020; Ghasemi-Mobtaker et al., 2020). Energy depletion and global warming potential caused by agriculture account for 6 and 17% of total Chinese energy depletion and global warming potential, respectively (Dong et al., 2008; Lin and Fei, 2015). In addition, a soil acidification and water pollution in agricultural areas are topics of major concern (Conley et al., 2009; Guo et al., 2010; Lee et al., 2020). LCA has been used to assess the net environmental impacts of major cereal crops and greenhouse vegetable production globally (Costa et al., 2020; Zhen et al., 2020). For example, the energy depletion in sunflower and pepper production systems are 27.0 and 20.3 GJ ha^-1^, respectively (Yousefi et al., 2017; Wang et al., 2018) and, according to Narh et al. (2020) the global warming potential from rice fields per season ranges from 0.86 to 1.71 t CO_2_ eq ha^-1^. Furthermore, surveys of citrus production have found that the average values of environmental risks indicated by acidification and eutrophication potential were 184 kg SO_2_ eq ha^-1^ and 110 kg PO_4_ eq ha^-1^, respectively (Yang et al., 2020). Studies using LCA methods have investigated the environmental impacts of fruit production systems but the situation in plum production systems remains poorly understood. Quantification of the environmental impacts of plum production may provide important insights and a basis for the evaluation environmental impacts of agriculture on a global scale.

The environmental impacts of agriculture production vary greatly, depending on nutrient management (Lenka et al., 2017) and farm size (Pishgar-Komleh et al., 2012). Recent robust evidence also shows that changes in environmental impacts are strongly responsive to different crop species and cropping systems (Zhang et al., 2016; Meng et al., 2019). Nutrient management is a major factor responsible for higher adverse environmental impacts. Mohammadi et al. (2010) investigated kiwifruit production and found that energy depletion of ~ 45% was generated by the total chemical fertilizer application. Chen et al. (2020) found that chemical fertilizers contribute > 90% of the total global warming potential from Chinese pomelo production. In addition, excessive chemical fertilizers are major pollutants that causes acidification and eutrophication (Grados and Schrevens, 2019). Hence, judicious fertilizer application is a fundamental step in ensuring high crop productivity in the long term (Yan et al., 2021).

Some studies have found that rational fertilizer management helps to achieve the “double-win” of increasing crop yields and simultaneously minimizing environmental impacts (Chen et al., 2014; Cui et al., 2018). However, traditional methods of determining the optimum nutrient supply to crops is complex and time consuming for farmers in developing countries, especially smallholder farmers. Effective methods are available to account for yield and environmental impacts, which can identify the most progressive farmers in a given area. For instance, these methods have been applied to pepper cultivation in southwest China and pomelo production in southeast China (Wang et al., 2018; Chen et al., 2020). Learning the management practices of the progressive farmers is an important step in establishing new advanced agricultural practices and reducing the environmental impacts of agricultural production. This method has been widely used in relation to pepper (Wang et al., 2018), citrus (Yang et al., 2020), and peach (Li et al., 2022) production. The use of farmer grouping can achieve robust results in developing countries due to its simplicity and low cost.

Smallholder farmers engaged in plum production often face significant management challenges, including the determination of optimal fertilizer application rates to achieve high fruit yields. To improve their management practices, quantifying the environmental impacts and estimating the total environmental index of plum production is imperative. Therefore, this study aimed to evaluate the environmental impacts of energy depletion, global warming potential, acidification potential, and eutrophication potential in plum production systems, and explore a local strategy for producing sustainable and eco-friendly plums.

## 2 Materials and methods

### 2.1 Survey region and data collection

**Figure 1:**
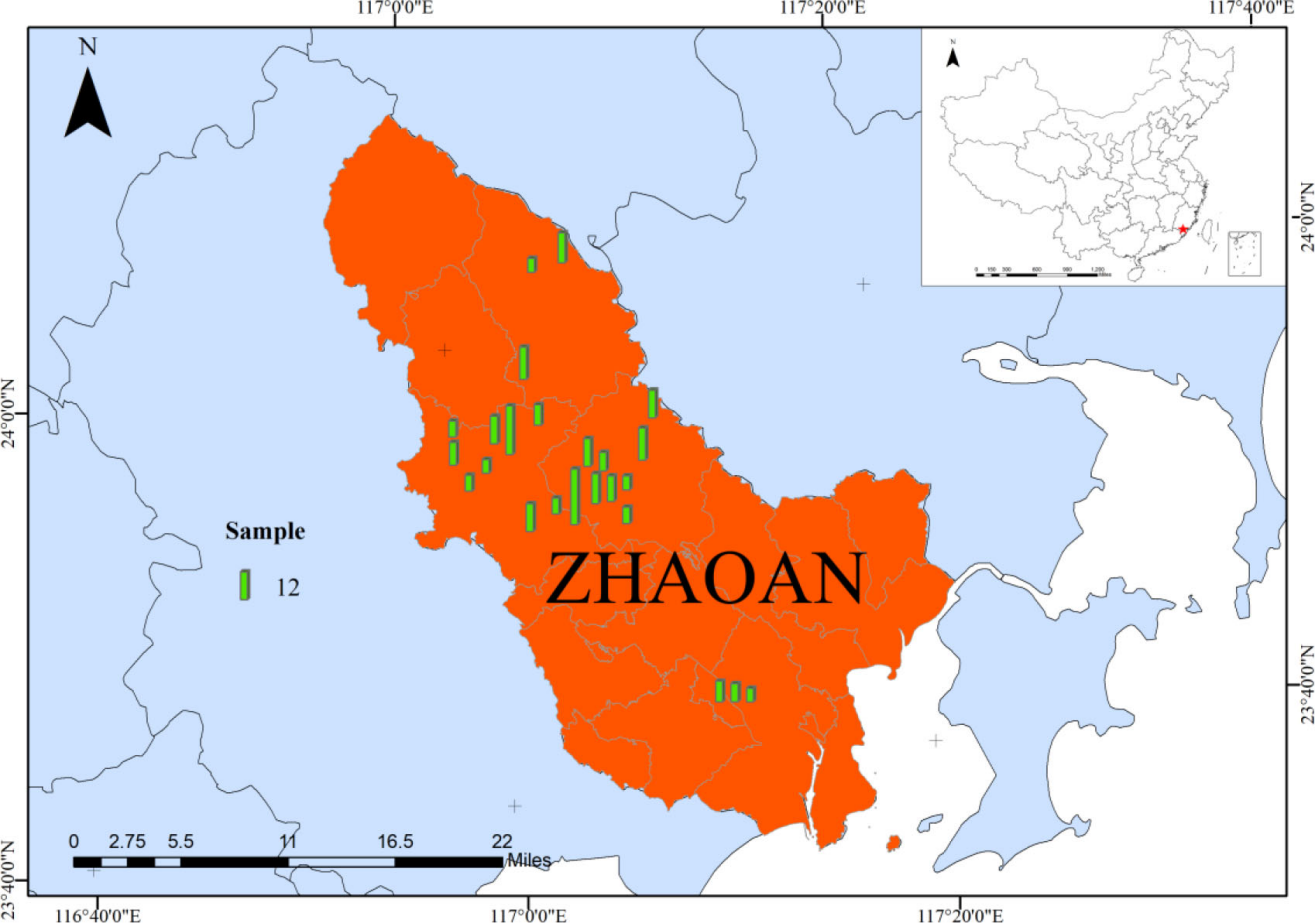
Geographical distribution of Zhao’an County, Fujian province, southeast China. The size of the bars in the figure represents the sample size.

The study area (23°35′-24°11′ N, 116°55′-117°22′ E) is in Zhao’an county, Fujian province, southeast China (**Figure 1**). It is characterized by a subtropical oceanic monsoon climate with an annual average temperature of 14.9-28.9°C and annual precipitation of ~ 1,148 mm. Here, twenty-four villages were randomly selected from four main plum production townships. Overall, 254 farmers were selected for a face-to-face interview in 2021. The survey aimed to obtain information on plum production such as varieties, plant densities, yields, fertilizer application rates, and pesticide and herbicide use.

### 2.2 Life cycle assessment

While sustainable smallholder agriculture has been a topic of extensive discussion and scientific exploration, there remains a lack of consensus on standardized approaches for evaluating sustainability (Guo et al., 2022). In this study, we quantitatively assessed the environmental impacts of plum production systems using the life cycle assessment (LCA) methodology, which follows the four-step framework outlined by the International Organization for Standardization (ISO 14040, 2006). This framework includes goal and scope definition, inventory analysis, impact assessment, and interpretation.

#### 2.2.1 Goal and scope definition

Here, four scenarios were defined for evaluating LCA environmental impacts, namely LL (low yield and low environmental impact), LH (low yield but high environmental impact), HL (high yield but low environmental impact), and HH (high yield and high environmental impact), whose comparison was one of the main objectives. The functional unit for the life cycle assessment was defined as one hectare of farmland with plum production for one year and a plum yield of one tonne.

The system boundary of the LCA was set using a cradle-to-market approach that extended from upstream planting to plum planting stage. The upstream stage of plum production comprised the production and transportation of materials (such as chemical fertilizers, farmyard manures, pesticides, and herbicides), and the plum planting stage included N losses (such as volatilization, runoff, and leaching losses) from farmyard manures and chemical fertilizers.

#### 2.2.2 Inventory analysis

Primary data collected from the on-site farm survey are listed in **Table 1**. Nutrient contents of farmyard manures are derived from data from the National Agricultural Technology Promotion Service Center (NATESC, 1999). Additionally, the equivalent coefficients of the emissions inventory for environmental impact potentials were obtained from data on energy consumption and pollutant emissions during the upstream stages of plum production based on the study of Wang et al. (2018). During the plum planting stage, N_2_O and NH_3_ emission data, and nitrogen and phosphorus loss data from farmyard manures and chemical fertilizers were collected from extant studies (Zhang et al., 2013; Zhang et al., 2017; Wang et al., 2018; Chen et al., 2020), and the mean values of the corresponding pollutant emission factors in these studies were used.

**Table 1:** Investigated inputs and outputs in the life cycle assessment of plum production in southeast China.

	Mean	Range	SD
Input			
Nitrogen (kg ha^-1^)	187.2	7.3~450	113.5
Phosphorus (kg ha^-1^)	81.7	3.2~197	49.5
Potassium (kg ha^-1^)	155.4	6.1~374	94.2
Farmyard manure (kg ha^-1^)	59.9	0~3000	322.7
Pesticides (kg ha^-1^)	0.7	0~9.0	0.9
Herbicide (kg ha^-1^)	20.4	7.5~78.8	12.3
Output			
Green plum yield (t ha^-1^)	20.1	1.7~97.5	15.3

#### 2.2.3 Impact assessment and interpretation

Environmental impacts considered were energy depletion (GJ), global warming (CO_2_ eq), acidification (SO_2_ eq), and eutrophication (PO_4_ eq) potential per unit area (in hectares, ha) in terms of the sum of the partial item equivalent of each input used. The various environmental impacts were estimated using the following equation (Wang et al., 2018):


$${\text{EI}}_{\text{j}}=\sum_{\text{i}=1}^{\text{n}}\left({\text{UP}}_{\text{ij}}+{\text{PS}}_{\text{ij}}\right)\times {\text{ Rate}}_{\text{i}}$$

Where EIj represents the impact category comprising GJ, CO_2_eq, SO_2_eq, and PO_4_eq potential per unit area (in hectares, ha). The emission potential of the j impact category per kg of i from the upstream of plum production stage was represented by UPij, while the emission potential of the j impact category per kg of i application at the plum planting stage was represented by PSij. The inputs used in plum production, such as chemical fertilizers, farmyard manures, pesticides, and herbicides, were represented by Ratei.

GJ, CO_2_ eq, SO_2_ eq, and PO_4_ eq potential per metric tonne (t) of plum production were calculated by the following equation:


$${\text{SEI}}_{\text{j}}=\frac{{\text{EI}}_{\text{j}}}{\text{Plum yield}}$$

Normalization values are generally the average levels of global energy consumption and environmental impacts. After normalization, various environmental impacts are of different importance to sustainable development, and need to be weighted. The normalization and weighting values of the four environmental impacts were obtained by Wang et al. (2014). The final total environmental index was calculated by the following equation:

Total environmental impact index


$$\;=\sum^{}\frac{{\text{SEI}}_{\text{j}}}{{\text{RV}}_{\text{j}}}\times {\text{W}}_{\text{j}}$$

Where SEIj represents the environmental impact potential of category j (in hectares, t). RVj is the relevant reference value of environmental impact j, and Wj is the weighting value of environmental impact j.

### 2.3 Fertilizer productivity

N partial fertilizer productivity (PFP_N_) is calculated as:


$${\text{PFP}}_{\text{N}}=\frac{\text{Plum yield}}{\text{N fertilizer input}}$$

### 2.4 Data analysis

Data processing was conducted using Microsoft Office Excel 2016, and all statistical analyses was conducted using the SPSS 21.0 software package. One-way analysis of variance and the least significant difference test (LSD) were used to check the differences of plum yield and environmental impacts per unit among the different groups.

## 3 Results

### 3.1 Inputs and yields in plum production

Plum production input and output data from the 254 farmers were collected and analyzed. In the study area the average nitrogen (N), phosphorus (P), and potassium (K) application rates in chemical fertilizers were 187.2, 81.7, and 155.4 kg ha^-1^, respectively, and the mean farmyard manure rate (range) was 59.9 kg ha^-1^ (0-3000 kg ha^-1^). The average pesticide input was 0-9.0 kg ha^-1^ and herbicide inputs were 7.5-78.8 kg ha^-1^. In addition, the average plum yield was ~ 20.1 t ha^-1^, ranging from 1.7 to 97.5 t ha^-1^ (**Table 1**).

### 3.2 Environmental impacts of plum production

Mean energy depletion, global warming, acidification, and eutrophication potentials were 18.17 GJ ha^-1^, 3.63 t CO_2_ eq ha^-1^, 42.18 kg SO_2_ eq ha^-1^, and 25.06 kg PO_4_ eq ha^-1^, respectively. Fertilizer N was a major locus of the environmental impact on plum production and was responsible for 52.06%, 85.67%, 98.99%, and 98.24% of the total energy depletion, global warming, acidification, and eutrophication potentials, respectively (**Table 2**). In addition, the average energy depletion, global warming, acidification, and eutrophication potentials per tonne of plum production were 1.42 GJ t^-1^, 0.28 t CO_2_ eq t^-1^, 3.21 kg SO_2_ eq t^-1^, and 1.90 kg PO_4_ eq t^-1^, respectively (**Table 3**).

**Table 2:** Environmental impacts per ha of land of plum production in southeast China.

Environmental impact category		Nitrogen	Phosphorus	Potassium	Farmyard manure	Pesticides	Herbicides	Total
Energy depletion	Mean (GJ ha^-1^)	9.46	0.94	2.75	0.14	4.86	0.02	18.17
	Percentage (%)	52.06	5.16	15.13	0.77	26.75	0.11	100.00
Global warming potential	Mean (t CO_2_ eq ha^-1^)	3.11	0.15	0.10	0.01	0.25	0.01	3.63
	Percentage (%)	85.67	4.13	2.75	0.28	6.89	0.28	100.00
Acidification potential	Mean (kg SO_2_ eq ha^-1^)	41.76	0.11	0.09	0.01	0.21	0	42.18
	Percentage (%)	98.99	0.26	0.21	0.02	0.50	0	100.00
Eutrophication potential	Mean (kg PO_4_ eq ha^-1^)	24.62	0.39	0.01	0	0.04	0	25.06
	Percentage (%)	98.24	1.56	0.04	0	0.16	0	100.00

**Table 3:** Environmental impacts per tonne of plum production in southeast China.

Environmental impact category		Nitrogen	Phosphorus	Potassium	Farmyard manure	Pesticides	Herbicides	Total
Energy depletion	Mean (GJ t^-1^)	0.71	0.07	0.21	0.01	0.42	0	1.42
	Percentage (%)	50.28	4.98	14.64	0.85	29.21	0.04	100.00
Global warming potential	Mean (t CO_2_ eq t^-1^)	0.24	0.01	0.01	0	0.02	0	0.28
	Percentage (%)	85.11	3.98	2.77	0.43	7.57	0.14	100.00
Acidification potential	Mean (kg SO_2_ eq t^-1^)	3.17	0.01	0.01	0	0.02	0	3.21
	Percentage (%)	98.93	0.27	0.21	0.02	0.57	0	100.00
Eutrophication potential	Mean (kg PO_4_ eq t^-1^)	1.87	0.03	0	0	0	0	1.90
	Percentage (%)	98.23	1.54	0.04	0.01	0.18	0	100.00

### 3.3 Environmental impacts of four groups of plum farmers

**Figure 2:**
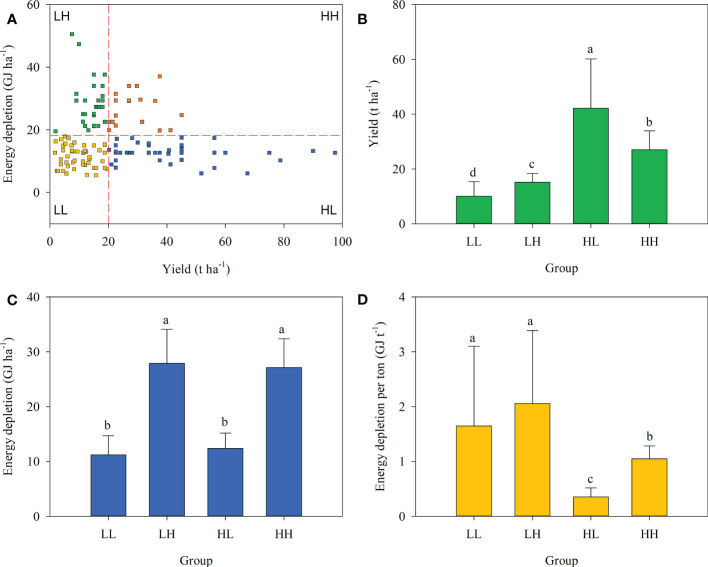
Farmer grouping based on plum yield and energy depletion. **(A)**, yield **(B)**, energy depletion per hectare **(C)**, and energy depletion per tonne of plum production **(D)** across four farmer groups.

**Figure 3:**
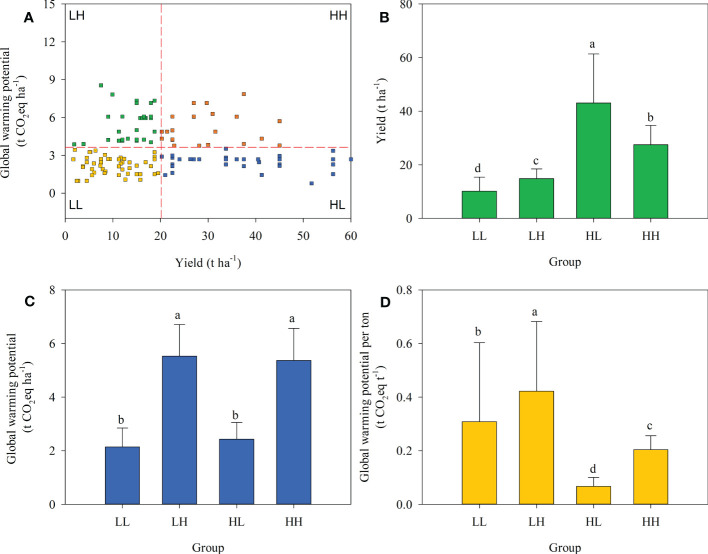
Farmer grouping based on plum yield and eutrophication potential **(A)**, yield **(B)**, eutrophication potential per hectare **(C)**, and eutrophication potential per tonne of plum production **(D)** across four farmer groups.

**Figure 4:**
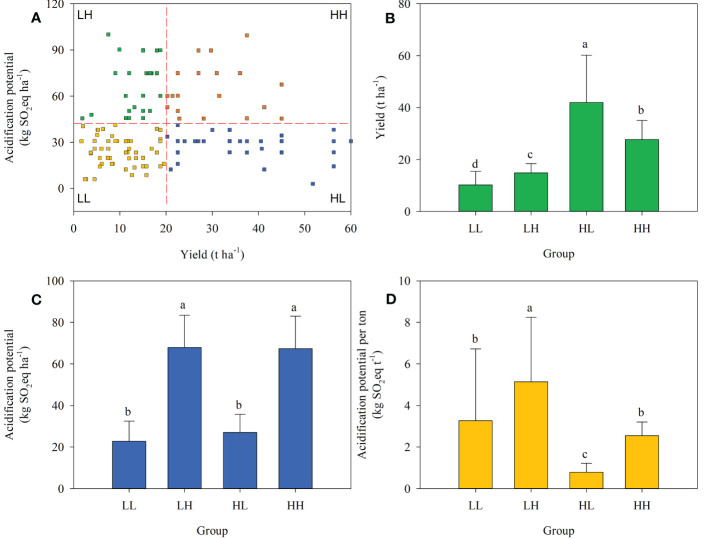
Farmer grouping based on plum yield and global warming potential **(A)**, yield **(B)**, global warming potential per hectare **(C)**, and global warming potential per tonne of plum production **(D)** across four farmer groups.

**Figure 5:**
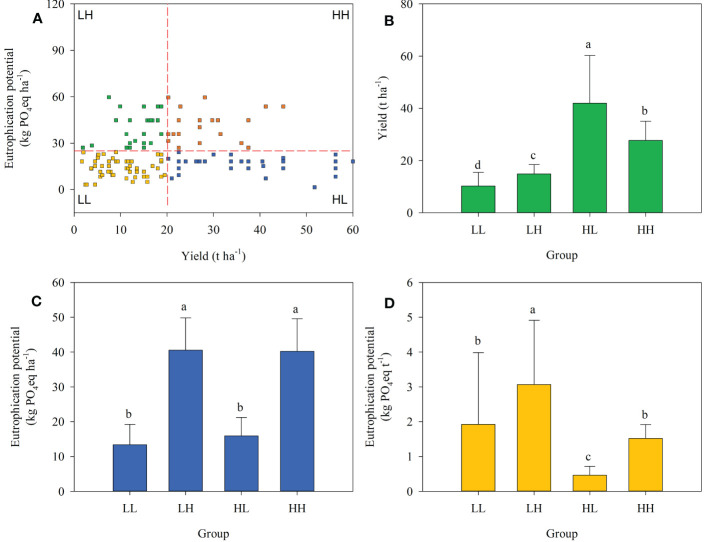
Farmer grouping based on plum yield and acidification potential **(A)**, yield **(B)**, acidification potential per hectare **(C)**, and acidification potential per tonne of plum production **(D)** across four farmer groups.

Overall, 19.7% of farmers were in the HL group, higher than in the HH group (13.3%) but lower than LL (39.0%) or LH (28.0%). Significant differences in energy depletion, global warming, acidification, and eutrophication potentials among the four groups were expressed either in terms of field area or on a yield production basis (**Figures 2**–**5**).

Considering energy depletion, the yields of LL, LH, HL, and HH groups were 10.02, 15.12, 42.15, and 27.03 t ha^-1^, respectively. When expressed on an area basis the energy depletion value of the HL group was 12.38 GJ ha^-1^ and was significantly lower than that of LH (27.87 GJ ha^-1^) or HH (27.11 GJ ha^-1^) groups. When expressed based on yield the energy depletion in the HL group was 0.35 GJ t^-1^ and significantly lower than in the other groups (**Figure 2**).

In terms of global warming potential, the yields of LL, LH, HL, and HH groups were 10.15, 14.82, 43.06, and 27.52 t ha^-1^, respectively. When expressed based on an area basis the global warming potential of the HL group was 2.43 t CO_2_ eq ha^-1^ and was significantly lower than that of LH (5.53 t CO_2_ eq ha^-1^) or HH (5.37 t CO_2_ eq ha^-1^) groups. When expressed based on yield the global warming potential in the HL group was 0.07 t CO_2_ eq t^-1^ and was significantly lower than in the other groups (**Figure 3**).

Similarly, acidification and eutrophication potentials were divided into four groups. The HL group had acidification and eutrophication potentials of 27.02 kg SO_2_ eq ha^-1^ and 15.94 kg PO_4_ eq ha^-1^, respectively, when measured on an area basis. When measured on a yield basis, the HL group had acidification and eutrophication potentials of 0.78 kg SO_2_ eq ha^-1^ and 0.46 kg PO_4_ eq ha^-1^, respectively, as shown in **Figures 4**, **5**.

**Figure 6:**
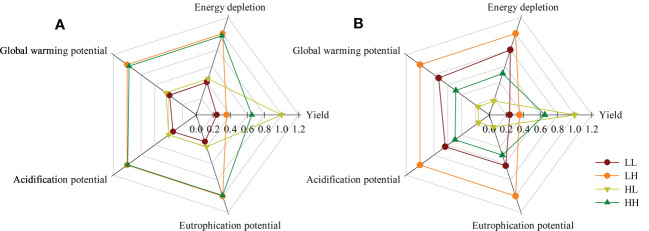
Relative comparison among the four groups based on different indicators (**A**: per ha, **B**: per tonne).

Overall, the plum yields of the HL group were 109-114% higher compared to the mean of all 254 farmer yields due to more advanced management practices. When expressed based on an area basis the energy depletion, global warming, acidification, and eutrophication potentials were 31.9, 33.1, 36.0, and 36.7% lower in this system. When expressed based on yield the energy depletion, global warming, acidification, and eutrophication potentials were 75.4, 75.0, 75.6, and 75.8% lower (**Figure 6**).

### 3.4 Environmental indices of plum production

During the normalization step, the four environmental impacts were ranked in descending order as eutrophication potential, acidification potential, global warming potential, and energy depletion. Eutrophication potential was identified as the primary environmental impact associated with the production of 1 tonne of plum fruit. During the weighting step the total environmental impact index of the HL group was 0.06, and this was 76.9, 85.7, and 71.4% lower than LL, LH, and HH groups, respectively (**Table 4**).

**Table 4:** Normalization and weighting of plum production per tonne of in environmental impacts.

Environmental impact category	Unit	Reference value	Normalization value				Weight		Total environmental index				
			LL	LH	HL	HH			LL	LH	HL	HH
Energy depletion	GJ t^-1^	2590	0.00064	0.00079	0.00014	0.00040	0.28		0.00018	0.00022	0.00004	0.00011
Global warming potential	t CO_2_eq t^-1^	6.87	0.04488	0.06140	0.00981	0.02968	0.23		0.01032	0.01412	0.00226	0.00683
Acidification potential	kg SO_2_eq t^-1^	52.26	0.06249	0.09838	0.01499	0.04871	0.26		0.01625	0.02558	0.00390	0.01266
Eutrophication potential	kg PO_2_eq t^-1^	1.88	1.02207	1.63117	0.24596	0.80840	0.23		0.23508	0.37517	0.05657	0.18593
							Total		0.26	0.42	0.06	0.21

### 3.5 Factor analysis in plum production

**Figure 7:**
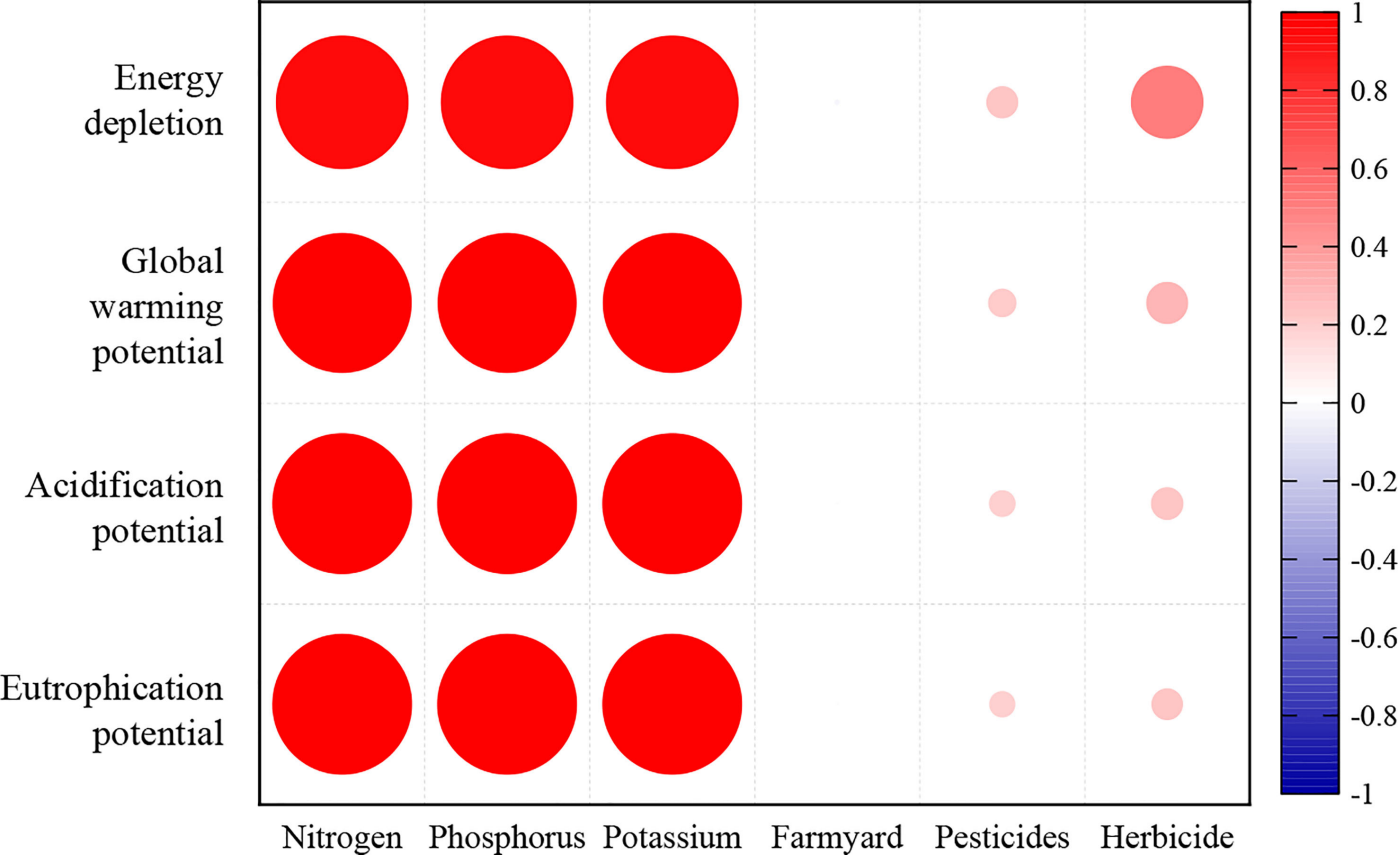
Correlations between agricultural material inputs and each environmental impact potential.

**Figure 8:**
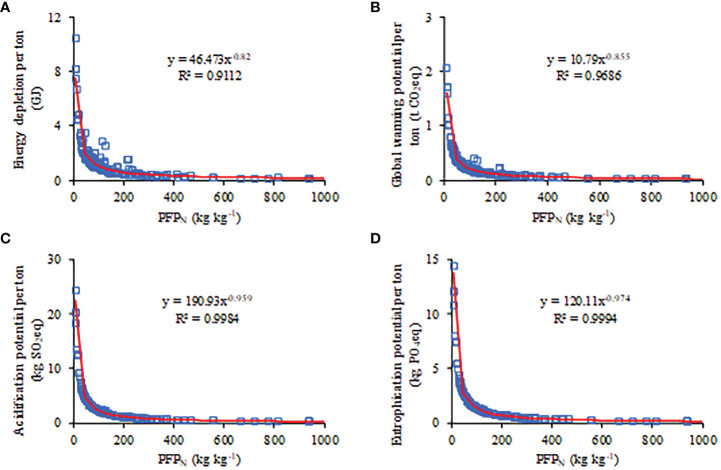
Correlation of energy depletion **(A)**, global warming potential **(B)**, acidification potential **(C)**, and eutrophication potential **(D)** per tonne with PFP_N_ for plum production.

The correlations between different agricultural inputs and each environmental impact were analyzed. **Figure 7** indicates that chemical fertilizer was significantly positively correlated with environmental impacts. The environmental impacts decreased as a power function with increasing PFP_N_. For example, when the PFP_N_ value reached 200 kg kg^-1^ the environmental impacts per tonne of plum production declined by ~ 61% compared with current management practices. Energy depletion, global warming, acidification, and eutrophication potentials per tonne of plum production decreased to 0.60 GJ t^-1^, 0.12 t CO_2_ eq t^-1^, 1.19 kg SO_2_ eq t^-1^, and 0.69 kg PO_4_ eq t^-1^, respectively (**Figure 8**).

## 4 Discussion

The environmentally sensitive development of agriculture is crucial, and the environmental impacts of agriculture have been of increasing concern in recent years (van der Werf et al., 2020). However, the life-cycle environmental impacts of plum production have not been extensively studied. This study evaluated a range of environmental impacts (including energy depletion, global warming potential, acidification potential, and eutrophication potential) of plum production systems and designed a suitable local strategy for the sustainable development of the plum industry. The study revealed that the environmental impact of plum production exceeded that of other orchard systems, such as mango and olive (Jekayinfa et al., 2013; Pergola et al., 2013). To mitigate these impacts, the effects of various nutrient management practices adopted by farmers were evaluated. Based on a farmer grouping method, the HL group implemented mitigation measures that maintained high plum yields while reducing environmental impacts. The most effective nutrient management practices were identified, and integrated N management practices in plum production were found to be feasible and necessary. It is worth noting that the study was conducted in a specific geographic area, and further research may be needed to determine if the findings are generalizable to other regions.

### 4.1 Benchmarking with other agricultural systems

Generally, fruit production exhibits higher environmental impacts than other crops, particularly cereals. A study in northeast Thailand by Soni et al. (2013) shows that the energy depletion in fruit production was 48%, 64%, and 89% higher than in rice, soybean, or maize, respectively. Previous studies also reveal that the contributions of rice and wheat production to Chinese agricultural GHG emissions decreased but those of fruits increased from 2001 to 2018 (Chen et al., 2021). Similar trends were observed in acidification and eutrophication potentials (Martin-Gorriz et al., 2020). Fruit crops produce much higher environmental impacts expressed on an area or yield basis than most other crops as a result of high application rates of urea and NPK compound fertilizers (Wu et al., 2021). Furthermore, summarizing previous studies on the environmental impacts of orchard systems clearly shows that the environmental impacts are higher in plum production systems than in those of other fruits (**Table 5**). These differences are mainly due to high application rates of fertilizers, especially fertilizer N (Chen et al., 2020). Here, N was the major factor responsible for higher environmental impacts and accounted ~ 83.74% of all environmental indices in the current study (**Table 2**). The average fertilizer N rate in plum production was ~ 187 kg ha^-1^, ~ 1.63 times (range 66.1-230.0 kg ha^-1^) more than previously reported in fruit orchards (**Table 5**). A rational fertilizer N management strategy is therefore a priority in decreasing the environmental impacts of plum production, especially in intensively managed cultivation systems.

**Table 5:** Environmental impacts of plum production (as determined in the current study) and of other fruit production systems (as determined by literature search).

Item/Orchard	Eenvironmental impacts (per ha)	Eenvironmental impacts (per t)	N rate (kg ha^-1^)	Reference
Energy depletion				
Plum	18.17	1.42	187.2	This study (calculated)
Apricot	20.00	0.90	80.0	Gezer et al., 2003
Canola	2.15	0.12	111.4	Mousavi-Avval et al., 2011
Mango	7.50	0.50	–	Jekayinfa et al., 2013
Peach	11.00	0.29	66.1	Royan et al., 2012
Tangerine	26.86	0.43	78.1	Mohammadshirazi et al., 2012
Global warming potential				
Plum	3.63	0.28	187.2	This study (calculated)
Apple	2.60	0.12	91.0	Aguilera et al., 2013
Citrus	2.60	0.24	–	Nemecek et al., 2012
Mango	0.40	0.04	–	Graefe et al., 2012
Passion	1.80	0.11	–	Graefe et al., 2012
Pineapple	2.30	0.06	–	Graefe et al., 2012
Acidification potential				
Plum	42.18	3.20	187.2	This study (calculated)
Almond	27.19	8.24	180.0	Bartzas et al., 2017
Apple	30.78	0.95	80.0	Bartzas et al., 2017
Pistachio	21.78	8.71	230.0	Bartzas et al., 2017
Eutrophication potential				
Plum	25.06	1.90	187.2	This study (calculated)
Almond	11.95	3.62	180.0	Bartzas et al., 2017
Apple	14.26	0.44	80.0	Bartzas et al., 2017
Pistachio	9.60	3.84	230.0	Bartzas et al., 2017

### 4.2 Changes required for sustainable plum production

Optimal application rates of N in agriculture production can provide economic and ecological benefits while also benefiting human health and welfare (Qiao et al., 2018). However, excessive use of fertilizer N does not increase yields and poses a significant environmental threat (Dong et al., 2020). Wang et al. (2020) previously demonstrated a 52% reduction potential in N inputs for pear orchards. A national survey conducted from 2005 to 2014 on N inputs and outputs showed that the N balance was positive in 97% of counties, with fruit production systems exhibiting the highest N surplus values, up to 429 kg ha^-1^ (Zhang et al., 2020). Lower fertilizer N application rates with high efficiency in fruit production have high potential to reduce energy depletion, global warming, soil acidification, and eutrophication potentials (Guo et al., 2021). Other high environmental impacts could also be achieved by lower N partial factor productivity (PFP_N_) values in plum production. In the present study, the plum PFP_N_ value was determined to be 107 kg kg^-1^. Wang et al. (2018) reported a decrease in mean energy depletion, global warming, acidification, and eutrophication potential by 35.5%, 37.3%, 33.9%, and 34.4%, respectively, when the PFP_N_ rate was increased from 49 to 73 kg kg^-1^.The current results also demonstrate that more efficient N management is vital in minimizing environmental impacts.

The farmer grouping method based on farm survey data is a simple method of identifying optimum N management strategies with high yields and low environmental impacts (Chen et al., 2020). For example, Ying et al. (2017) reported a 12% yield increase in wheat yields together with a 54% decline in potential N losses based on the grouping method. This method also performs well in the efficiency analysis of sugarcane production systems in Thailand (Ullah et al., 2019). Here, the large variation in yields and environmental impacts of plum production raised the possibility of applying this grouping method. In the cultivation of plums, previous experts recommended a nitrogen fertilizer application rate of 191 kg ha^-1^ (Hu, 2019). Limited by the socioeconomic situation, the previous recommendation system focused only on the yield effects, but currently realizing sustainable development has become a global priority. Irrational fertilizer management occurs and further design schemes are necessary in plum production. Integrated soil crop system management practices and innovative management programme (integrated knowledge and product strategies) have been investigated as potential approaches for achieving more precise and efficient N management in agricultural production, with promising results that demonstrate a “double-win” situation. Chen et al. (2014) demonstrated higher fertilizer N efficiency in cereals to produce more grain with low environmental costs, and the optimum fertilizer N application rates for rice, wheat, and maize were 146, 192, and 214 kg ha^-1^, respectively. Wang et al. (2021) report that integrated N management in vegetable production may also decrease the N application rate by 38% compared with farming N management practices. Consistent with previous studies, improved N management strategies also mitigate the environmental impacts of plum production. Optimizing fertilizer N application rates may result in lower N losses and pollution risks, and this is required to alleviate anthropogenically induced environmental impacts in orchard systems.

### 4.3 Outlook and limitations

Continuing efforts are being made towards narrowing the yield and efficiency gaps through the exploitation of sustainable management strategies (Chen et al., 2014). However, the use of farmer grouping is a direct and efficient way of determining the plum management strategy from the local farmers in the HL group, resulting in higher yields in plum orchards and decreasing environmental impacts. The farmers in the HL group may be more receptive to rapid acquisition, updating and application of innovative N management practices than those in the other groups, as has been found in previous studies of citrus-producing regions in southwest China (Yang et al., 2020). Similar results have been obtained in peach production, in which farmers in the HL group used 46% less fertilizer than those in the LH group (Li et al., 2022). It is crucial to maximize yields while minimizing associated environmental harm. However, the popularization and application of this technology still faces many challenges. It is not a simple task to change the habits of smallholder farmers (Helmizar et al., 2019), and there is an opportunity for social science research to contribute to agronomic efforts and help to promote sustainable agricultural development.

Moreover, there were several limitations in the present study. First, despite the use of site-specific empirical models to estimate N losses, there are unavoidable uncertainties associated with the process. N losses are influenced by various factors, including fertilizer N application rate, soil type, climatic conditions, and type of fertilizer N applied. Therefore, even when using empirical parameters, accurately estimating N losses can be challenging due to the heterogeneity of the region (Chien et al., 2009). Second, fertilization based on the “4 Rs” (right rate, right source, right time, and right place) is key nutrient management to sustaining crop productivity (Mikkelsen, 2011). A rational fertilizer N application rate based on the HL group in plum production can produce high yields and low environmental impacts. However, the right source, right time, and right place remain poorly understood and require further study.

## 5 Conclusions

The findings of this study highlight the significant environmental risks associated with intensive plum production in southeast China. The results showed that there were considerable variations in the potential environmental impacts (such as energy depletion, global warming potential, acidification potential, and eutrophication potential) among the four farmer groups, which were mainly attributed to differences in yields and environmental impacts. The HL group achieved a significantly higher plum yield than the mean of all 254 farmers, mainly due to better management practices, resulting in a mitigation potential of > 70% for the total environmental impact index. This achievement was mainly due to lower fertilizer N rates. By adopting the optimal management strategy of the HL group, farmers at the county level can narrow the differences in yields and environmental impacts, resulting in high yields and low environmental impacts simultaneously. Therefore, this study provides valuable information for achieving sustainable plum fruit production in southeast China.

## Data availability statement

The raw data supporting the conclusions of this article will be made available by the authors, without undue reservation. Please contact the corresponding author for access to the original data.

## Author contributions

XY: Conceptualization, investigation, writing-original draft, formal analysis, visualization. DY: Conceptualization, writing-original draft. YT: Conceptualization, writing-original draft. MM: Writing-reviewing & editing. PC: Writing-reviewing & editing. CT: Formal analysis. WX: Data curation. BS: Data curation. JX: Data curation. JZ: Supervision, resources, funding acquisition. All authors contributed to the article and approved the submitted version.

## References

[B1] Aguilera E. Lassaletta L. Gattingerd A. Gimenoe B. (2013). Managing soil carbon for climate change mitigation and adaptation in Mediterranean cropping systems: A meta-analysis. Agric. Ecosyst. Environ. 168, 25–36. doi: 10.1016/j.agee.2013.02.003

[B2] Bartzas G. Vamvuka D. Komnitsas K. (2017). Comparative life cycle assessment of pistachio, almond and apple production. Info. Proc. Agric. 4, 188–198. doi: 10.1016/j.inpa.2017.04.001

[B3] Carranca C. Brunetto G. Tagliavini M. (2018). Nitrogen nutrition of fruit trees to reconcile productivity and environmental concerns. Plants 7 (1), 4. doi: 10.3390/plants7010004 29320450 PMC5874593

[B4] Chen X. Cui Z. Fan M. Vitousek P. Zhao M. Ma W.. (2014). Producing more grain with lower environmental costs. Nature 514, 486–489. doi: 10.1038/nature13609 25186728

[B5] Chen X. Ma C. Zhou H. Liu Y. Huang X. Wang M.. (2021). Identifying the main crops and key factors determining the carbon footprint of crop production in china 2001-2018. Resour. Conserv. Recy. 172, 105661. doi: 10.1016/j.resconrec.2021.105661

[B6] Chen X. Xu X. Lu Z. Zhang W. Yang J. Hou Y.. (2020). Carbon footprint of a typical pomelo production region in China based on farm survey data. J. Clean Prod. 277, 124041. doi: 10.1016/j.jclepro.2020.124041

[B7] Chien S. Prochnow L. Cantarella H. (2009). Recent developments of fertilizer production and use to improve nutrient efficiency and minimize environmental impacts. Adv. Agron. 102, 267–322. doi: 10.1016/S0065-2113(09)01008-6

[B8] Conley D. Paerl H. Howarth R. Boesch D. Seitzinger S. Havens K.. (2009). Controlling eutrophication: Nitrogen and phosphorus. Science 323, 1014–1015. doi: 10.1126/science.1167755 19229022

[B9] Costa M. Chadwick D. Saget S. Rees R. Williams M. Styles D. (2020). Representing crop rotations in life cycle assessment: A review of legume LCA studies. Int. J. Life Cycle Assess. 25, 1942–1956. doi: 10.1007/s11367-020-01812-x

[B10] Cui Z. Zhang H. Chen X. Zhang C. Ma W. Huang C.. (2018). Pursuing sustainable productivity with millions of smallholder farmers. Nature 555, 363–366. doi: 10.1038/nature25785 29513654

[B11] Dong H. Li Y. Tao X. Peng X. Li N. Zhu Z. (2008). China Greenhouse gas emissions from agricultural activities and its mitigation strategy. Trans. Chin. Soc Agric. Eng. 24, 269–273.

[B12] Dong Y. Zeng F. Yuan J. Zhang G. Chen Y. Liu X.. (2020). Integrated rice management simultaneously improves rice yield and nitrogen use efficiency in various paddy fields. Pedosphere 30 (6), 863–873. doi: 10.1016/S1002-0160(20)60042-X

[B13] FAO (2021). The state of food security and nutrition in the world 2021 (Rome: Food and Agriculture Organization of the United Nations).

[B14] Gezer I. Acaroğlu M. Haciseferoğullari H. (2003). Use of energy and labour in apricot agriculture in Turkey. Biomass Bioenergy 24, 215–219. doi: 10.1016/S0961-9534(02)00116-2

[B15] Ghasemi-Mobtaker H. Kaab A. Rafiee S. Ghasemi-Mobtaker H. Kaab A. Rafiee S. (2020). Application of life cycle analysis to assess environmental sustainability of wheat cultivation in the west of Iran. Energy 193, 116768. doi: 10.1016/j.energy.2019.116768

[B16] Grados D. Schrevens E. (2019). Multidimensional analysis of environmental impacts from potato agricultural production in the Peruvian central Andes. Sci. Total Environ. 663, 927–934. doi: 10.1016/j.scitotenv.2019.01.414 30739860

[B17] Graefe S. Tapasco J. Gonzalez A. (2012). Resource use and GHG emissions of eight tropical fruit species cultivated in Colombia. Fruits 68 (4), 303–314. doi: 10.1051/fruits/2013075

[B18] Guo J. Liu X. Zhang Y. Shen J. Han W. Zhang W.. (2010). Significant acidification in major Chinese croplands. Science 327, 1008–1010. doi: 10.1126/science.1182570 20150447

[B19] Guo X. Wang C. Zhang F. (2022). Construction of an index system for sustainability assessment in smallholder farming systems. Front. Agr. Sci. Eng. 9 (4), 511–522. doi: 10.15302/J-FASE-2022463

[B20] Guo X. Zhao D. Zhuang M. Wang C. Zhang F. (2021). Fertilizer and pesticide reduction in cherry tomato production to achieve multiple environmental benefits in guangxi, China. Sci. Total Environ. 793, 148527. doi: 10.1016/j.scitotenv.2021.148527 34174594

[B21] Helmizar H. Yuswita E. Putra A. (2019). Analysis of the nutrients and microbiological characteristics of the Indonesian dadih as a food supplementation. Glob. J. Health Sci. 11, 155–161. doi: 10.5539/gjhs.v11n1p155

[B22] Hu Z. (2019). Analysis on the characteristics of fertilizer demand and fertilization management of green plum. Mod. Agric. Sci. Technolo. 24, 70–71.

[B23] ISO 14040 (2006). Environmental management-life cycle assessment-principles and framework (Geneva, Switzerland: International Organization for Standardization).

[B24] Jekayinfa S. Adebayo A. Afolayan S. Daramola E. (2013). On-farm energetics of mango production in Nigeria. Renew. Energy 51, 60–63. doi: 10.1016/j.renene.2012.09.004

[B25] Lee E. Zhang X. Adler P. Kleppel G. Romeiko X. (2020). Spatially and temporally explicit life cycle global warming, eutrophication, and acidification impacts from corn production in the U.S. Midwest. J. Clean. Prod. 242, 118465. doi: 10.1016/j.jclepro.2019.118465

[B26] Lenka S. Lenka N. Singh A. Singh B. Raghuwanshi J. (2017). Global warming potential and greenhouse gas emission under different soil nutrient management practices in soybean-wheat system of central India. Environ. Sci. pollut. Control Ser. 24, 4603–4612. doi: 10.1007/s11356-016-8189-5 27957695

[B27] Li Z. Chen Y. Meng F. Shao Q. Heal M. Ren F.. (2022). Integrating life cycle assessment and a farmer survey of management practices to study environmental impacts of peach production in Beijing, China. Environ. Sci. pollut. R. 29, 57190–57203. doi: 10.1007/s11356-022-19780-0 35344146

[B28] Li Y. Yang M. Zhang Z. Li W. Guo C. Chen X.. (2019). An ecological research on potential for zero-growth of chemical fertilizer use in citrus production in China. Ekoloji 28, 1049–1059.

[B29] Lin B. Fei R. (2015). Regional differences of CO_2_ emissions performance in china’s agricultural sector: A malmquist index approach. Eur. J. Agron. 70, 33–40. doi: 10.1016/j.eja.2015.06.009

[B30] Loiseau E. Aissani L. Le Féon S. Laurent F. Cerceau J. Sala S.. (2018). Territorial life cycle assessment (LCA): What exactly is it about? a proposal towards using a common terminology and a research agenda. J. Clean. Prod 176, 474–485. doi: 10.1016/j.jclepro.2017.12.169

[B31] Martin-Gorriz B. Gallego-Elvira B. Maestre-Valero J. Martínez-Alvarez V. (2020). Life cycle assessment of fruit and vegetable production in murcia region (south-east Spain) and evaluation of impact mitigation practices. J. Clean. Prod. 265, 121656. doi: 10.1016/j.jclepro.2020.121656

[B32] Meng W. He M. Li H. Hu B. Mo X. (2019). Greenhouse gas emissions from different plant production system in China. J. Clean. Prod. 235, 741–750. doi: 10.1016/j.jclepro.2019.07.009

[B33] Mikkelsen R. (2011). The "4R" nutrient stewardship framework for horticulture. HortTechnology 21 (6), 658–662. doi: 10.21273/HORTTECH.21.6.658

[B34] Mohammadi A. Rafiee S. Mohtasebi S. Rafiee H. (2010). Energy inputs–yield relationship and cost analysis of kiwifruit production in Iran. Renew. Energ. 35 (5), 1071–1075. doi: 10.1016/j.renene.2009.09.004

[B35] Mohammadshirazi A. Akram A. Rafiee S. Avval S. Kalhor E. (2012). An analysis of energy use and relation between energy inputs and yield in tangerine production. renew. sustain. Energy Rev. 16, 4515e4521. doi: 10.1016/j.rser.2012.04.047

[B36] Mousavi-Avval S. Rafiee S. Jafari A. Mohammadi A. (2011). Energy flow modeling and sensitivity analysis of inputs for canola production in Iran. J. Clean. Prod. 19, 1464–1470. doi: 10.1016/j.jclepro.2011.04.013

[B37] Narh S. Darko D. Koranteng S. Tettey A. Agyei K. Acquah D. (2020). Quantifying greenhouse gas emissions from irrigated rice production systems in Ghana. J. Environ. Prot. 11 (11), 938. doi: 10.4236/jep.2020.1111059

[B38] NATESC (1999). Nutrients of organic fertilizers in China (Beijing: Science and Technology of China Press).

[B39] Nemecek T. Weiler K. Plassmann K. Schnetzer J. Gaillard G. Jefferies D.. (2012). Estimation of the variability in global warming potential of worldwide crop production using a modular extrapolation approach. J. Clean. Prod. 31, 106–117. doi: 10.1016/j.jclepro.2012.03.005

[B40] Pergola M. Favia M. Palese A. Perretti B. Xiloyannis C. Celano G. (2013). Alternative management for olive orchards grown in semi-arid environments: An energy, economic and environmental analysis. Sci. Hortic. 162, 380–386. doi: 10.1016/j.scienta.2013.08.031

[B41] Pishgar-Komleh S. Ghahderijani M. Sefeedpari P. (2012). Energy consumption and CO_2_ emissions analysis of potato production based on different farm size levels in Iran. J. Clean. Prod. 33, 183–191. doi: 10.1016/j.jclepro.2012.04.008

[B42] Qiao C. Xu B. Han Y. Wang J. Wang X. Liu L.. (2018). Synthetic nitrogen fertilizers alter the soil chemistry, production and quality of tea: a meta-analysis. Agron. Sustain. Dev. 38, 10. doi: 10.1007/s13593-017-0485-z

[B43] Royan M. Khojastehpour M. Emadi B. Ghasemi-Mobtaker H. (2012). Investigation of energy inputs for peach production using sensitivity analysis in Iran. Energy Convers. Manage. 64, 441–446. doi: 10.1016/j.enconman.2012.07.002

[B44] Shah F. Wu W. (2019). Soil and crop management strategies to ensure higher crop productivity within sustainable environments. Sustainability 11, 1485. doi: 10.3390/su11051485

[B45] Soni P. Taewichit C. Salokhe V. (2013). Energy consumption and CO_2_ emissions in rainfed agricultural production systems of northeast Thailand. Agr. Syst. 116, 25–36. doi: 10.1016/j.agsy.2012.12.006

[B46] Ullah A. Silalertruksa T. Pongpat P. (2019). Efficiency analysis of sugarcane production systems in Thailand using data envelopment analysis. J. Clean. Prod. 238, 117877. doi: 10.1016/j.jclepro.2019.117877

[B47] van der Werf H. Knudsen M. Cederberg C. (2020). Towards better representation of organic agriculture in life cycle assessment. Nat. Sustain. 3, 419–425. doi: 10.1038/s41893-020-0489-6

[B48] Wang X. Dou Z. Shi X. Zou C. Liu D. Wang Z.. (2021). Innovative management programme reduces environmental impacts in Chinese vegetable production. Nat. Food 2, 47–53. doi: 10.1038/s43016-020-00199-0 37117651

[B49] Wang C. Li X. Gong T. Zhang H. (2014). Life cycle assessment of wheat-maize rotation system emphasizing high crop yield and high resource use efficiency in quzhou county. J. Clean Prod. 68, 56–63. doi: 10.1016/j.jclepro.2014.01.018

[B50] Wang J. Zhang L. He X. Zhang Y. Wan Y. Duan S.. (2020). Environmental mitigation potential by improved nutrient managements in pear (*Pyrus pyrifolia* l.) orchards based on life cycle assessment: A case study in the north China plain. J. Clean Prod. 262, 121273. doi: 10.1016/j.jclepro.2020.121273

[B51] Wang X. Zou C. Zhang Y. Shi X. Liu J. Fan S.. (2018). Environmental impacts of pepper (*Capsicum annuum* l) production affected by nutrient management: A case study in southwest China. J. Clean. Prod. 171, 934–943. doi: 10.1016/j.jclepro.2017.09.258

[B52] Wu H. MacDonald G. Galloway J. Zhang L. Gao L. Yang L.. (2021). The influence of crop and chemical fertilizer combinations on greenhouse gas emissions: A partial life-cycle assessment of fertilizer production and use in China. Resour. Conserv. Recy. 168, 105303. doi: 10.1016/j.resconrec.2020.105303

[B53] Yan X. Chen X. Ma C. Cai Y. Cui Z. Chen X.. (2021). What are the key factors affecting maize yield response to and agronomic efficiency of phosphorus fertilizer in China? Field Crop Res. 270, 108221. doi: 10.1016/j.fcr.2021.108221

[B54] Yang M. Long Q. Li W. Wang Z. He X. Wang J.. (2020). Mapping the environmental cost of a typical citrus-producing county in China: Hotspot and optimization. Sustainability 12, 1827. doi: 10.3390/su12051827

[B55] Ying H. Ye Y. Cui Z. Chen X. (2017). Managing nitrogen for sustainable wheat production. J. Clean. Prod. 162, 1308–1316. doi: 10.1016/j.jclepro.2017.05.196

[B56] Yousefi M. Khoramivafa M. Damghani A. (2017). Water footprint and carbon footprint of the energy consumption in sunflower agroecosystems. Environ. Sci. pollut. R. 24, 19827–19834. doi: 10.1007/s11356-017-9582-4 28685342

[B57] Zhang Q. Chu Y. Xue Y. Ying H. Chen X. Zhao Y.. (2020). Outlook of china's agriculture transforming from smallholder operation to sustainable production. Glob. Food Secur. 26, 100444. doi: 10.1016/j.gfs.2020.100444

[B58] Zhang W. Dou Z. He P. Ju X. Powlson D. Chadwick D.. (2013). New technologies reduce greenhouse gas emissions from nitrogenous fertilizer in China. Proc. Natl. Acad. Sci. U.S.A. 110 (21), 8375e8380. doi: 10.1073/pnas.1210447110 23671096 PMC3666697

[B59] Zhang G. Lu F. Huang Z. Chen S. Wang X. (2016). Estimations of application dosage and greenhouse gas emission of chemical pesticides in staple crops in China. J. Appl. Ecol. 27, 2875–2883. doi: 10.13287/j.1001-9332.201609.031 29732850

[B60] Zhang D. Shen J. Zhang F. Li Y. Zhang W. (2017). Carbon footprint of grain production in China. Sci. Rep. 7 (1), 4126. doi: 10.1126/science.aao6621 28663590 PMC5491493

[B61] Zhen H. Gao W. Jia L. Qiao Y. Ju X. (2020). Environmental and economic life cycle assessment of alternative greenhouse vegetable production farms in peri-urban Beijing, China. J. Clean. Prod. 269, 122380. doi: 10.1016/j.jclepro.2020.122380

